# Immunomodulatory effects of complex probiotics on the immuno-suppressed mice induced by cyclophosphamide

**DOI:** 10.3389/fmicb.2023.1055197

**Published:** 2023-01-27

**Authors:** Weiwei Ma, Wenwen Li, Shuang Yu, Hongsheng Bian, Yanyan Wang, Yang Jin, Zhenhua Zhang, Qing Ma, Lili Huang

**Affiliations:** College of Pharmacy, Heilongjiang University of Chinese Medicine, Harbin, China

**Keywords:** complex probiotics, cyclophosphamide, immunoregulation, cytokine, gut microbiota

## Abstract

**Introduction:**

Previous studies have reported the beneficial effects of *Bifidobacterium animalis* subsp. *lactis* XLTG11, *Lacticaseibacillus casei* Zhang, and *Lactiplantibacillus plantarum* P8, respectively. However, studies on the immunomodulatory enhancing effects of three complex probiotics have not been conducted. The aim of our study is to investigate the immunomodulatory effects of complex probiotics effect on the immunosuppressed mice induced by cyclophosphamide (CTX).

**Methods:**

An immunocompromised mouse model was established by intraperitoneal injection of cyclophosphamide, which was gavage of different doses of complex probiotics and levamisole hydrochloride. The splenic and thymic indices, intestinal barrier, leukocyte and lymphocyte counts, percentage of splenic lymphocyte subpopulations, cytokine levels, and gut microbiota were determined.

**Results:**

Results showed that the complex probiotics significantly elevated the spleen and thymus indices, increased the villi and crypt depth and the goblet cells. The leukocyte and lymphocyte counts and the percentage of splenic lymphocyte subpopulations in the CTX-treated mice were significantly elevated by the complex probiotics. In addition, the cytokines (IL-6, IL-10, IL-1β, and IFN-γ) were significantly increased after complex probiotic treatment. The complex probiotics restored the gut microbiota structure to the pattern of the control group by reducing the ratio of Firmicutes/Bacteroidetes and enhancing the relative abundances of specific microbiota that produced short-chain fatty acids.

**Discussion:**

This study provides theoretical support for the immunity-enhancing function of the complex probiotics as well as a pharmacological basis for its further development and utilization.

## 1. Introduction

The novel coronavirus pneumonia (COVID-19) outbreak broke out globally in late 2019, and the immune system is a widely talked about topic in the context of the outbreak. Studies have shown that decreased immune function of the body is one of the important reasons for the development of COVID-19 (Jayawardena et al., 2020). Therefore, it is especially important to strengthen the immunity of the organism, improve the antiviral capacity and avoid the development of underlying diseases in oneself. Among the immunity-regulating drugs, chemical drugs have a limited role, obvious side effects and no specific efficacy in immunosuppressive diseases and viral infectious diseases (Hutchings et al., 2019). Therefore, attention should be paid to improve immunity in daily life, such as balanced diet, a reasonable mix of various nutrients, as well as supplemental intake of micro-ecological preparations, fermented dairy products, etc.

Probiotics has been gaining more and more attention in clinical use because of its safety, reliability and excellent performance (Zhang and Chen, 2018). *Bifidobacterium* spp. and *Lactobacillus* spp. are currently used for the development of immune enhancing functions of the body (Dan et al., 2019). *Lactobacillus* is an important component of the innate immune system of the organism, which is indispensable for maintaining the stable state of the immune function and plays a vital role in maintaining the host microecological balance and improving the function of the immune system of the organism. *Lactobacillus plantarum* can activate Th1 immune response (Kawashima et al., 2011), promote IgA secretion and prevention of influenza virus infection (Kikuchi et al., 2014), enhancement of the cytokine profile against mite allergy (Rigaux et al., 2009), and increase natural killer cell activity (Rizzo et al., 2013). *Bifidobacteria* are the main probiotics in the intestinal microbiota and play key roles in the intestinal micro-ecosystem, such as enhancing immunity (De Vuyst and Leroy, 2011; Hill et al., 2017; Wang et al., 2021). Shang et al. (2020) reported that the administration of *Bifidobacterium bifidum* H3-R2 could improve immune cell activity, balance expression of inflammatory cytokines and enhance the production of sIgA. The study found that daily consumption of *Bifidobacterium animalis* subsp. *lactis* HN019 enhances NK cell tumoricidal activity and PMN phagocytic capacity in healthy elderly adults, improving immunity (Lehtoranta et al., 2017).

It was shown that the *B*. *lactis* XLTG11, *L. casei* Zhang and *L. plantarum* P8 could enhance immune function in mice, respectively (Wang et al., 2013, 2014; Fu et al., 2020; Weiwei et al., 2022; Xu et al., 2022). However, studies on enhancing effects of complex probiotic preparations containing *B*. *lactis* XLTG11, *L. casei* Zhang and *L. plantarum* P8 have not been reported. Cyclophosphamide (CTX), a classical myelotoxic agent, has been used in previous study to establish an experimental model applicable to the evaluation of immunomodulation by antibiotics in normal and immunocompromised mice (Jimenez-Valera et al., 2003). Therefore, this study investigated the effect of different doses of complex probiotics on the immune function in the immunosuppressed mice induced by cyclophosphamide. This study will provide a scientific basis for the development and utilization of probiotic complex microecological preparations.

## 2. Materials and methods

### 2.1. Microbial strain and culture conditions

Complex probiotic powder used in this experiment is made of *B*. *lactis* XLTG11, *L. casei* Zhang and *L. plantarum* P8 in a ratio of 4: 3:3, provided by Jinhua Galaxy Biotechnology Co., Ltd. Complex probiotic powder was stored in the refrigerator at −80°C in different doses for reserve, and normal saline was used as the solvent for immediate use.

### 2.2. Animal care and handling

Male five-week-old BALB/c mice (body weight 20 ± 2 g) were purchased from the Liaoning Changsheng biotechnology co., Ltd. (SCXK<Liao>2020–0001). All mice were housed in a room under controlled temperature and humidity ranges of 22–24°C and 30–60%, respectively, on 12 h light–dark cycle and free access to food and water. After 1 week of acclimatization, mice were randomly divided into six groups with eight mice each: normal control (CTRL) group, CTX-treated group (Jiangsu Hengrui Pharmaceutical Co. Ltd., China), low dose of complex probiotics (CL) (2.5 × 10^6^ CFU day), medium dose of complex probiotics (CM) (5 × 10^6^ CFU day), high doses of complex probiotics (CH) (1 × 10^7^ CFU day) and levamisole hydrochloride (LEV)-treated group (Shanxi Taiyuan Pharmaceutical Co. Ltd., China). During the experiment, the CTRL and CTX groups were gavaged with saline, and the other four groups were gavaged with different concentrations complex probiotics and levamisole hydrochloride (10 mg/kg. bw) for 28 days, respectively. On the 23th and 24th, all mice except for the CTRL group were treated with an intraperitoneal injection of CTX (40 mg/kg. bw) once a day, while the CTRL group was treated with same dose of saline. After 30 min treatment of administration or saline on the 28th, fresh feces from each mouse of six group were collected immediately, and stored at −80°C until further analysis. At the end of the experiment, the blood and ileum tissue were obtained for next experiments (Zhou et al., 2021). All animal procedures were performed in accordance with the Guidelines for Care and Use of Laboratory Animals of Heilongjiang Chinese Medicine University and the experiments were approved by the Animal Ethics Committee of Heilongjiang Chinese Medicine University (ethic approval code: 2021121201).

### 2.3. Thymus and spleen index

The thymus and spleen were dissected and weighed to calculate the thymus and spleen index. Thymus or spleen index (mg/g) = thymus or spleen mass (mg)/animal body mass (g).

### 2.4. Histological analysis

The ileum intestinal tissues were prepared for histological analysis using the methods described by Meng et al. (2019) with some modification. In brief, the ileum tissue 2–3 cm was fixed in 4% paraformaldehyde for 48 h, after pruning, xylene dehydration, paraffin embedding, and then sliced in 4 μm thickness.

In order to measure villus height and crypt depth, tissue was stained with hematoxylin and eosin (HE) after deparaffinization. The images were taken by a light microscope (Nikon Eclipse Ci-L, 100× magnification, Japan). The top of the villus to the crypt transition was counted as villus length, while the crypt depth was recorded as the invagination between two villi. The intestinal villus length and crypt depth were measured in each sample *via* Image Pro Plus 6.0 software (Media Cybernetics, United States).

In order to measure quantities of goblet cells, tissue was stained with Alcian blue periodic acid Schiff staining kit (AB-PAS) (Servicebio, Wuhan, China) after deparaffinization. The images were taken by a light microscope (Nikon Eclipse Ci-L, 100× magnification Japan). The total quantities of goblet cells in epithelial cells of the ileum tissues were counted by Image pro plus software 6.0.

### 2.5. Hematological analysis

Blood sample was collected and added with EDTA-2 K for anti-coagulation. The leukocyte and lymphocyte counts were detected by a hematology analyzer (XS-500i, Sysmex, Shanghai, China).

### 2.6. Splenic lymphocyte subpopulation percentage test

The mouse spleen was isolated from the mice and used to extract lymphocytes according to the method of Cai et al. (2022). Briefly, anti-CD3, anti-CD4, anti-CD8 and anti-NK surface antibodies were added to the cell suspension at a certain dilution ratio and incubated for 30 min at 4°C, without light. Next, the lymphocytes were centrifuged at 4°C for 5 min and the supernatant was discarded, then resuspended with 1 ml PBS for flow cytometry analysis (Becton, Dickinson and Company, United States). FITC, APC, PE and PE/Cyanine7 were used for analysis with 1 × 10^5^ cells on board. The results were expressed as the percentage of CD3^+^, CD4^+^CD8^−^, CD4-CD8^+^T lymphocytes and natural killer cells (NK cells) of the composite probiotic on the subpopulation of splenic lymphocytes in mice.

### 2.7. Serum level testing

After the last administration, the whole blood was centrifuged (3,000 *g*, 10 min) to extract the serum, which was finally frozen in the refrigerator at −80°C for measurement. The ELISA kit for serum IL-6, IL-10, IL-1β, and IFN-γ (Jiangsu Zymeon Industrial Co. Ltd) were used to determine the cytokine level according to the instructions.

### 2.8. Gut microbiota analysis

DNA was extracted from fecal samples of each group (*n* = 8) by the E.Z.N.A.® Stool DNA Kit according to the manufacturer’s recommendations, and DNA purity and concentration were detected by 1% agarose gel electrophoresis, then taken appropriate samples diluted to 1 ng/μL in sterile water in a centrifuge tube (Guan et al., 2021). The microbiota composition was assessed by PCR targeting the V3-V4 region of the bacterial 16S rRNA gene with the primer 343F-798R (fwd 5′-TACGGRAGGCAGCAG-3′ and rev 5′-AGGGTATCTAATCCT-3′) (Nossa et al., 2010). The PCR products were purified by AMPure XT beads (Beckman Coulter Genomics, Danvers, MA, United States) and quantified by Qubit (Invitrogen, United States). Samples were sequenced on an Illumina Miseq platform (Illumina Inc., San Diego, CA, United States) according to standard protocols. The raw data was merged with Flash (V1.2.11) software and filtered by QIIME (V1.8.0) to collect the high-quality clean tags (Caporaso et al., 2010; Reyon et al., 2012). The valid tags were clustered by UCHIME (version 2.4.2) into OTUs of ≥97% similarity (Edgar et al., 2011). OTUs were analyzed by RDP classifier Naive Bayesian classification algorithm, and annotated with taxonomic information (Wang et al., 2007).

### 2.9. Statistical analysis

SPSS 26.0 statistical software was used to analyze all data, and the results were expressed as means ± standard. Using one-way analysis of variance (ANOVA), *p* < 0.05 was considered statistically significant.

## 3. Results

### 3.1. Effects of complex probiotics on the thymus and spleen index

**Figure 1:**
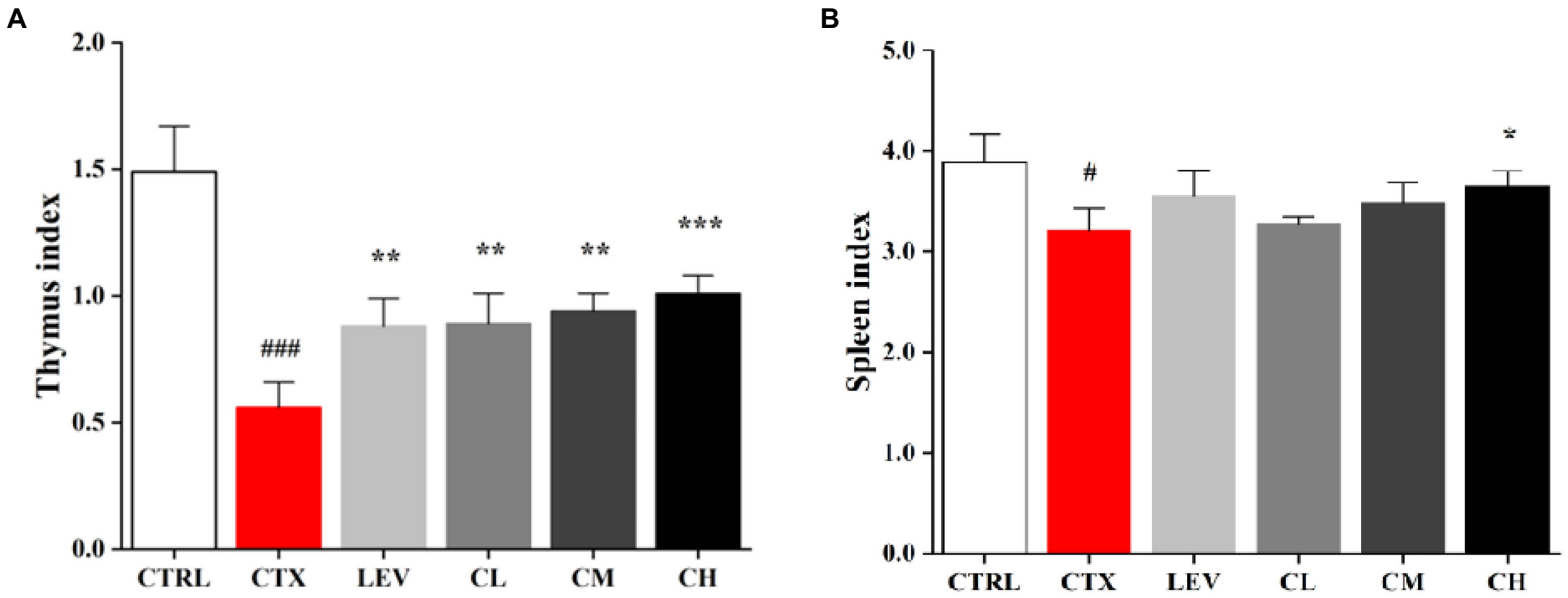
Complex probiotics adjusted immune organ indices in CTX-induced immunosuppression mice. **(A)** Thymus index. **(B)** Spleen index. CTRL, normal control group; CTX, model control group; LEV, levamisole hydrochloride; CL, low-dose complex probiotics; CM, Medium-dose complex probiotics; CH, high-dose complex probiotics. All data are expressed as mean ± SD. ^#^*p* < 0.05, ^##^*p* < 0.01, ^###^*p* < 0.001 compared with CTRL; ^*^p < 0.05, ^**^p < 0.01, ^***^p < 0.001 compared with CTX.

As shown in Figure 1, compared with the CTRL group, the thymus and spleen indexes of the CTX group were markedly reduced (*p* < 0.001), indicating the immunosuppressed mice model was successfully constructed. As compared of CTX group, the thymus indexes of different dose of the complex probiotics groups were increased significantly (*p* < 0.01), especially high-dose complex probiotics (*p* < 0.001). However, there was no significant difference in spleen index between complex probiotics group and CTX group except CH group (*p* < 0.05).

### 3.2. Effects of complex probiotics on gut barrier disruption

**Figure 2:**
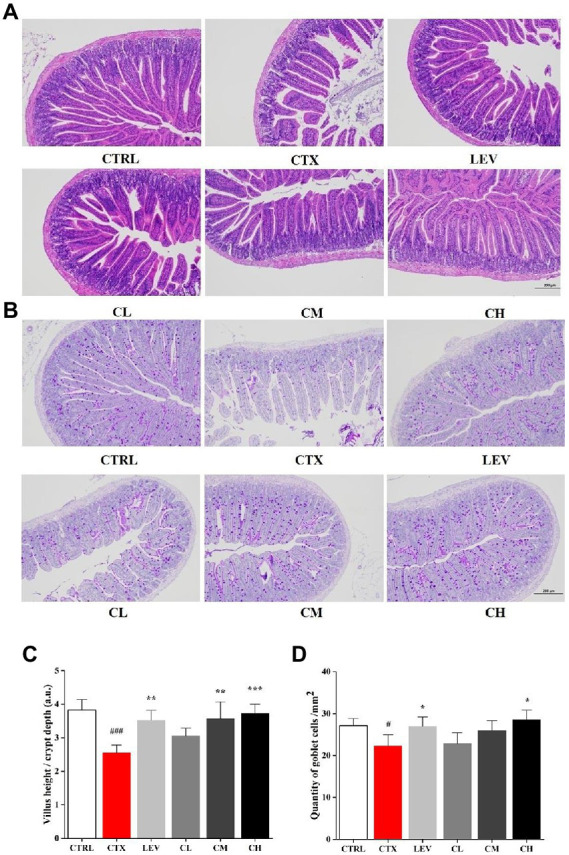
Complex probiotics treatments relived intestinal immune disorder in CTX-induced immunosuppression mice. (*n* = 8 for each group). **(A)** HE staining of jejunum section (100×, Scale bar = 200 mm). **(B)** AB-PAS staining of jejunum section. (100×, Scale bar = 200 mm). **(C)** The ratio of villus height/crypt depth. **(D)** The quantity of goblet cells/mm^2^. CTRL, normal control group; CTX, model control group; LEV, levamisole hydrochloride; CL, low-dose complex probiotics; CM, Medium-dose complex probiotics; CH, high-dose complex probiotics. All data are expressed as mean ± SD. ^#^*p* < 0.05, ^##^*p* < 0.01, ^###^*p* < 0.001 compared with CTRL; ^*^p < 0.05, ^**^p < 0.01, ^***^p < 0.001 compared with CTX.

The ileum histological changes in each group are shown in Figures 2A,C. In the CTRL group, the small intestinal mucosa tissue structure was intact, and the villi were arranged neatly and closely with uniform thickness, but the intestinal mucosa tissue was damaged, intestinal wall was thinned, villi were atrophied in different lengths and arranged sparsely, the inflammatory cells infiltrated in CTX group. The damage of small intestinal mucosa tissue was improved to different degrees, and the villus lesions were reduced in LEV group and probiotics treated groups, compared to CTX group. Additionally, the villus height (VH)/crypt depth (CD) ratio in the CTX group were lower than that in the CTRL group, which was significantly increased in the LEV (*p* < 0.01), CM (*p* < 0.01) and CH (*p* < 0.001) groups. The AB-PAS stains of ileum sections are shown in Figures 2B,D, which have the similar tendency toward HE stain of ileum sections. The goblet cells in the CTX group were significantly (*p* < 0.05) lower than that in the CTRL group, indicating that the intestine structural integrity was destroyed by CTX. However, compared to CTX group, the high dose of the complex probiotics treatment significantly increased (*p* < 0.05) ileum goblet cells, which was similar with the LEV group.

### 3.3. Effects of complex probiotics on leukocyte and lymphocyte count

**Figure 3:**
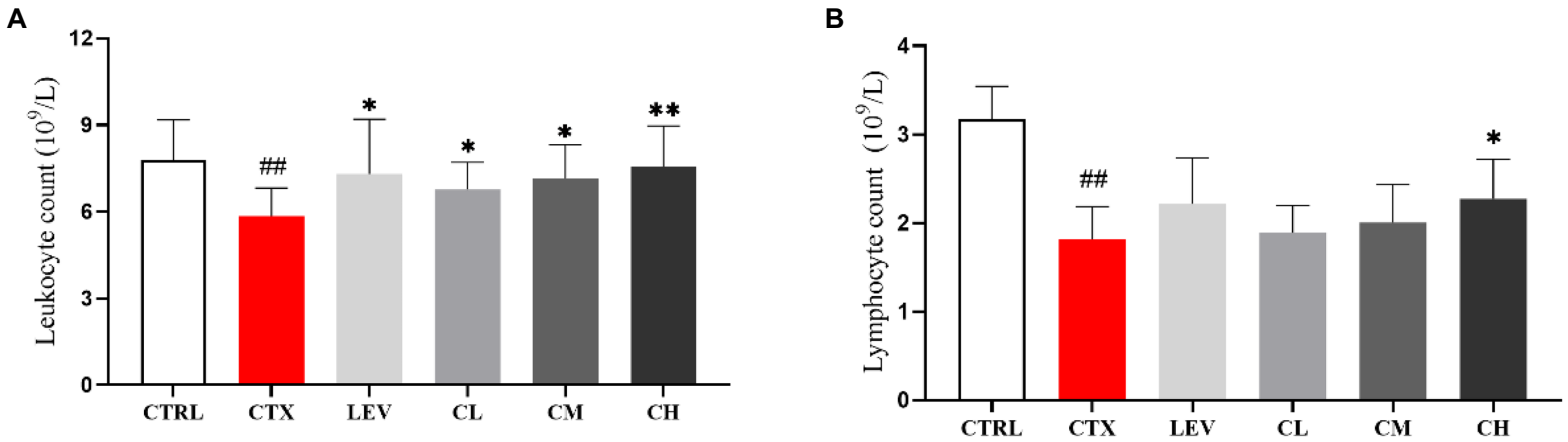
Complex probiotics adjusted **(A)** leukocyte count and **(B)** lymphocyte count in CTX-induced immunosuppression mice. (*n* = 8 for each group). CTRL, normal control group; CTX, model control group; LEV, levamisole hydrochloride; CL, low-dose complex probiotics; CM, Medium-dose complex probiotics; CH, high-dose complex probiotics. All data are expressed as mean ± SD. ^#^*p* < 0.05, ^##^*p* < 0.01, ^###^*p* < 0.001 compared with CTRL; ^*^p < 0.05, ^**^p < 0.01, ^***^p < 0.001 compared with CTX.

As shown in Figure 3, leukocyte and lymphocyte count were significantly (*p* < 0.01) reduced by CTX-treatment when compared to the CTRL group. The leukocyte and lymphocyte count of the LEV, CM, and CH groups were significantly increased (*p* < 0.05, *p* < 0.05, *p* < 0.01) compared with the CTX group, but there was no significant change in the CL group (*p* > 0.05). The lymphocyte count was significantly (*p* < 0.05) increased in the CH group, whereas no significant changes were found in the LEC, CL, and CM groups.

### 3.4. Effects of complex probiotics on the percentage of splenic lymphocyte subpopulations in mice

**Figure 4:**
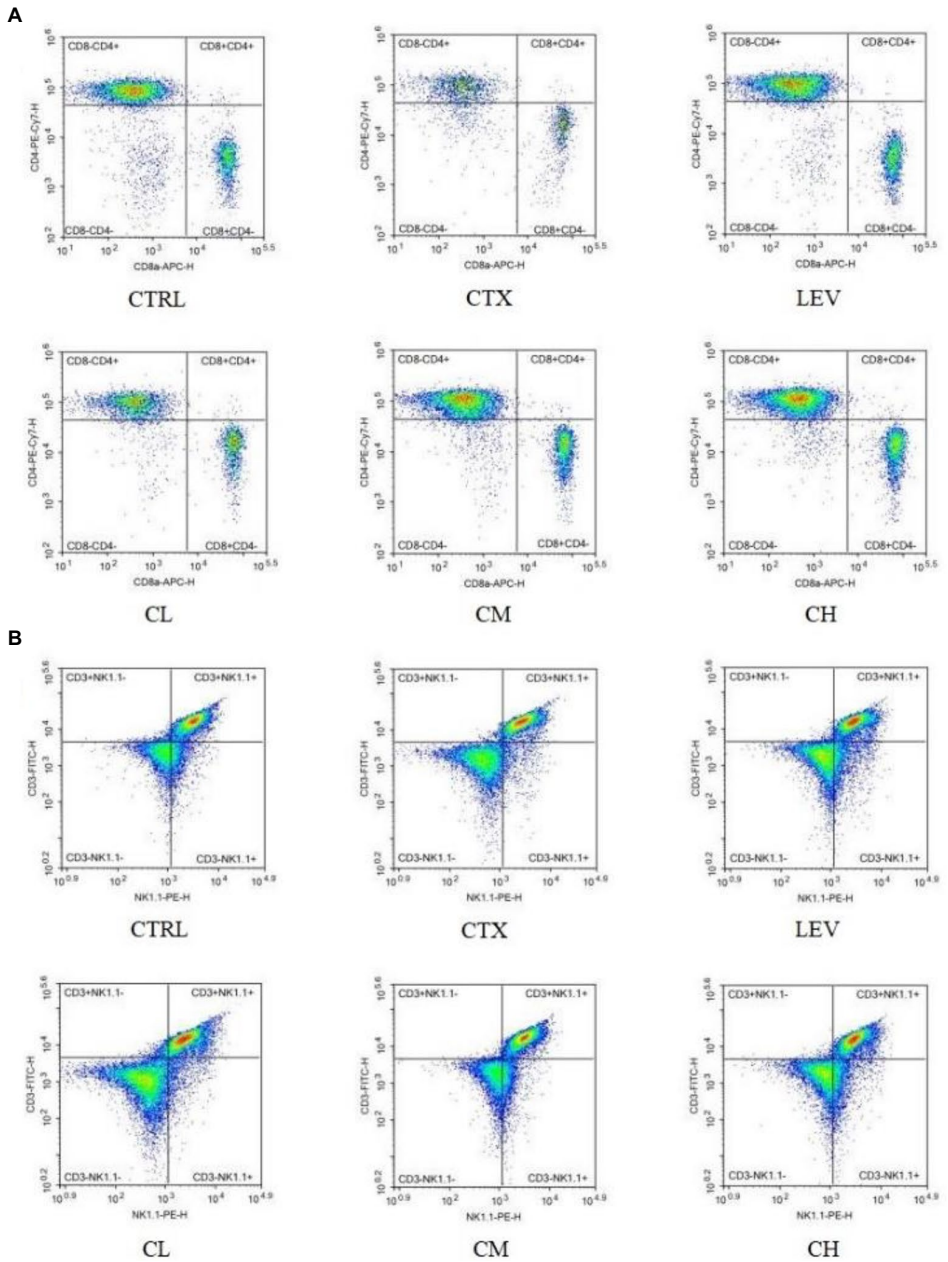
Percentage of splenic lymphocyte subsets of mice in various groups detected by flow cytometry. **(A)** CD4^+^CD8^−^ and CD4^−^CD8^+^ T lymphocytes, **(B)** CD3^+^ and NK cells.

As shown in Table 1; Figure 4, the percentages of splenic lymphocytes CD3^+^, CD4^+^CD8^−^, CD4^−^CD8^+^ and NK cells were reduced in the CTX group compared with the CTRL group (*p* < 0.01, *p* < 0.05, *p* < 0.01, *p* < 0.01). Compared with the CTX group, splenic lymphocyte CD3^+^, CD4^+^CD8^−^, CD4^−^CD8^+^ were significantly (*p* < 0.01) increased in the LEV, CL, CM, and CH groups. The percentage of NK cells significantly increased in the LEV (*p* < 0.05) and CH (*p* < 0.01) groups, respectively.

**Table 1:** Effect of complex probiotics on percentage of splenic lymphocyte subsets of CTX mice.

Group	CD3^+^T	CD4^+^CD8^−^	CD4^−^CD8^+^	NK
CTRL	41.97 ± 6.51	68.33 ± 4.82	24.98 ± 4.14	11.18 ± 3.03
CTX	32.02 ± 4.25^**^	64.48 ± 3.20^*^	20.26 ± 2.62^**^	7.08 ± 1.49^**^
LEV	40.64 ± 8.30^##^	71.65 ± 1.79^###^	23.67 ± 2.52^#^	10.18 ± 3.85^#^
CL	42.64 ± 5.48^##^	69.98 ± 3.58^##^	23.20 ± 1.90^#^	8.29 ± 2.54
CM	45.67 ± 6.41^###^	71.77 ± 3.60^###^	24.66 ± 2.39^##^	9.52 ± 3.16
CH	49.52 ± 4.22^###^	72.16 ± 2.62^###^	24.87 ± 2.04^##^	11.08 ± 2.57^##^

CTRL, normal control group; CTX, model control group; LEV, levamisole hydrochloride; CL, low-dose complex probiotics; CM, Medium-dose complex probiotics; CH, high-dose complex probiotics. (−x ± S, *n* = 8). ^#^*p* < 0.05, ^##^*p* < 0.01, ^###^*p* < 0.001 compared with CTRL; *p < 0.05, **p < 0.01, ***p < 0.001 compared with CTX.

### 3.5. Effect of complex probiotics on serum cytokine levels in mice

**Figure 5:**
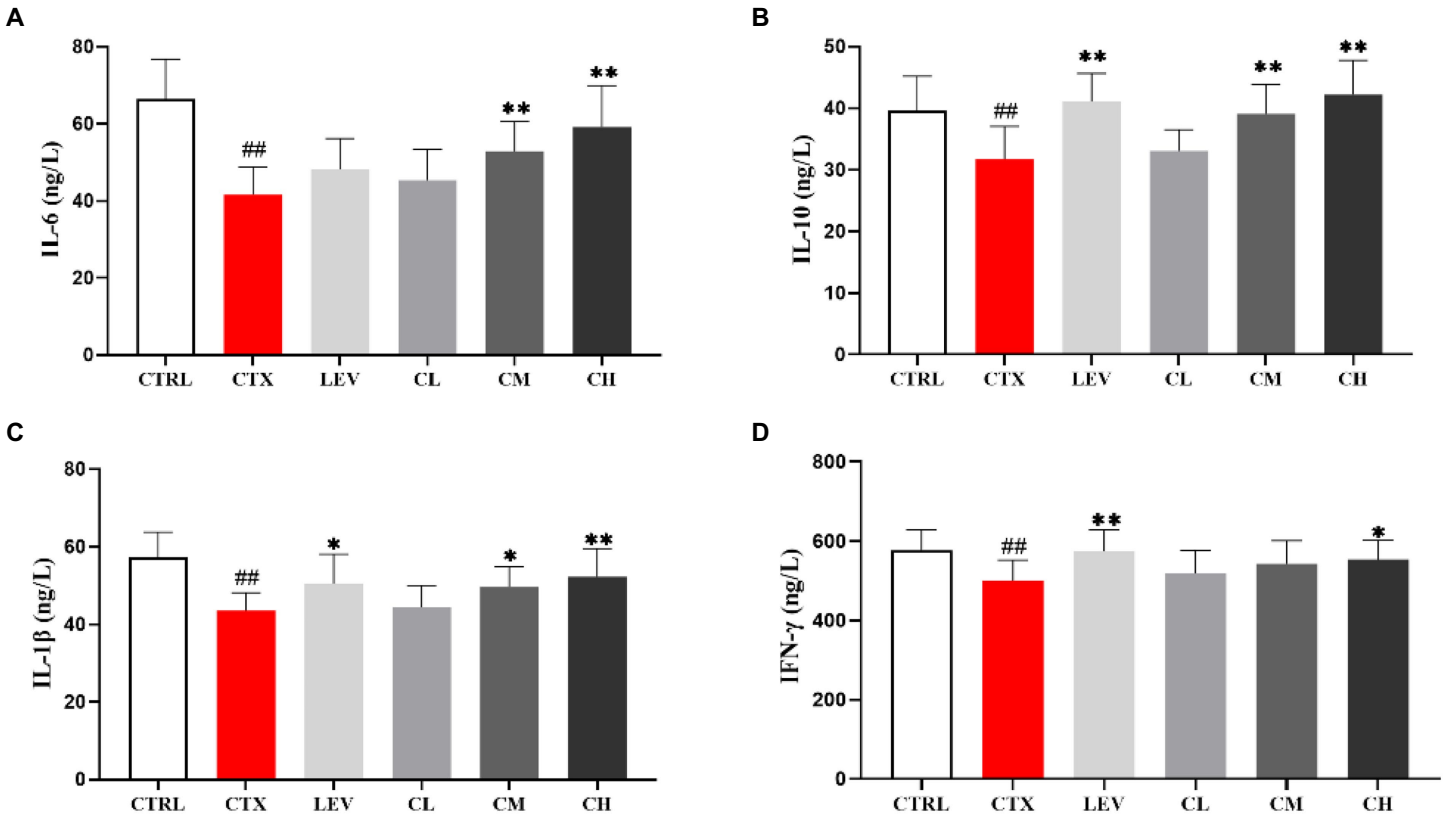
Effect of complex probiotics on serum cytokine levels in mice. (*n* = 8 for each group). **(A)** IL-6, **(B)** IL-10, **(C)** IL-1β, and **(D)** IFN-γ. CTRL, normal control group; CTX, model control group; LEV, levamisole hydrochloride; CL, low-dose complex probiotics; CM, Medium-dose complex probiotics; CH, high-dose complex probiotics. All data are expressed as mean ± SD. ^#^*p* < 0.05, ^##^*p* < 0.01, ^###^*p* < 0.001 compared with CTRL; ^*^p < 0.05, ^**^p < 0.01, ^***^p < 0.001 compared with CTX.

As shown in Figure 5, compared with the CTRL group, serum IL-6, IL-10, IL-1β and IFN-γ levels in the CTX group were significantly decreased (*p* < 0.01). However, IL-6 levels in the CM and CH groups were significantly increased (*p* < 0.01), IL-10 levels in the LEV, CM and CH groups were significantly increased (*p* < 0.01), IL-1β levels in the LEV, CM and CH groups were significantly increased (*p* < 0.05, *p* < 0.05, and *p* < 0.01), IFN-γ levels in the LEV and CH groups were significantly increased (*p* < 0.01 and *p* < 0.05) when compared to the CTX group.

### 3.6. Gut microbiota analysis

**Figure 6:**
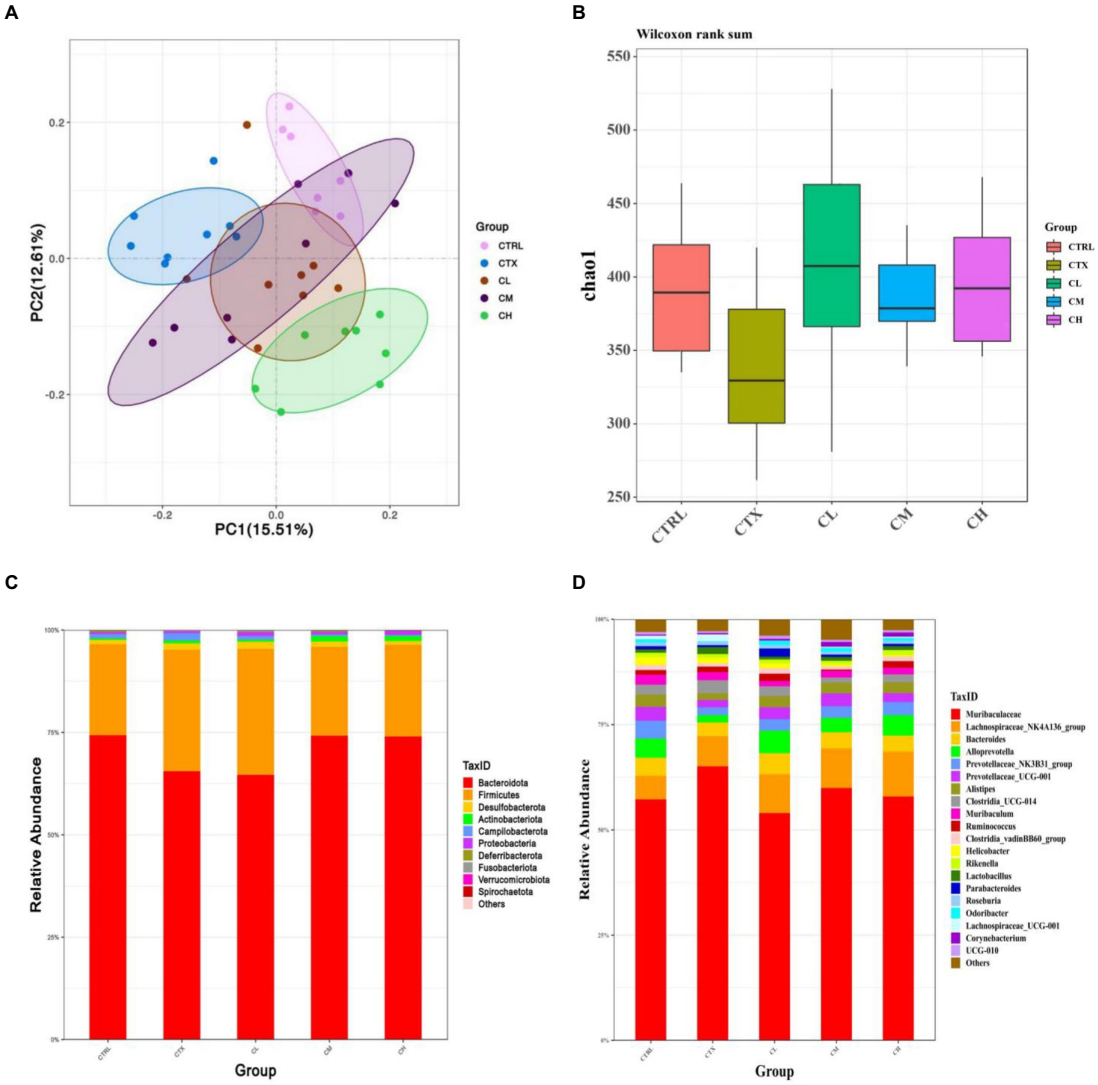
Complex probiotics treatments attenuated gut microbiota dysbiosis in CTX-induced immunosuppression mice. (*n* = 8 for each group). **(A)** Chao1 indexes. **(B)** Binary Jaccard-based PCoA. **(C)** Bacterial taxonomic profiling at the phylum level. **(D)** Bacterial taxonomic profiling at the genus level. CTRL, normal control group; CTX, model control group; LEV, levamisole hydrochloride; CL, low-dose complex probiotics; CM, medium-dose complex probiotics; CH, high-dose complex probiotics.

In order to detect the influence of complex probiotics on the structure and composition of the intestinal flora in the CTX mice, we collected the feces of five groups of mice and performed the 16S rDNA sequencing to analyze gut microbiota. As shown in Figure 6A. The Chao1 index was decreased by CTX-treatment in relative to CTRL group. After complex probiotics treatment, the Chao1 index of the CM and CH groups was reversed when compared with the CTX group. To visualize the overall gut microbiota structure, the β-diversity of microbial composition was calculated using Binary Jaccard-based PCoA (Figure 6B). It was obviously separated among the CTX group and the other groups, especially the CH group.

We analyzed the relative abundances of phylum (Figure 6C) and genus (Figure 6D). At the phylum level, more than 90 percent of the microbiota among all groups was composed by Bacteroidetes and Firmicutes. In the CTX group, Bacteroidetes was decreased, but Campilobacterota and Verrucomicrobia were increased when compared to the CTRL group. After treatment with complex probiotics, the relative abundances of Firmicutes decreased, therefore, the Firmicutes/Bacteroidetes (F/B) ratio was lower than that of the other groups. However, the relative abundance of Bacteroidetes was increased in the CM and CH groups, the relative abundances of Campilobacterota and Verrucomicrobia were reduced in the CM and CH groups, which was to similar to those in the CTRL group.

At the genus level, in the CTX group, the relative abundances of *Alloprevotella*, *Prevotellaceae*_NK3B31_group, *Prevotellaceae*_UCG-001, *Alistipes, Clostridia*_vadinBB60_group, *Helicobacter*, *Odoribacter* were decreased when compared to the CTRL group. The relative abundances of *Lactobacillus* and *Lachnospiraceae*_UCG-001 was increased in CTX group compared to the CTRL group. The relative abundances of *Alloprevotella*, *Clostridia*_vadinBB60_group, *Alistipes* and *Odoribacter* were increased in the CH group when compared to the CTX group. *Lachnospiraceae*_UCG-001 was decreased in the complex probiotic groups. Furthermore, *Lachnospiraceae*_NK4A136_group and *Corynebacterium* were increased in the CM and CH group compared with the CTX group.

## 4. Discussion

Probiotics are active microbial agents that are beneficial to the host and contribute to the ecological balance of the gut microbiota. When consumed in sufficient doses, they stimulate the host’s immune system and enhance the host’s immune response, thus helping the host to resist various diseases, which is extremely important for the improvement of human health (Ezendam and van Loveren, 2006). It has been reported that the global probiotic market is growing at an annual rate of 15–20%, and the relationship between probiotics and human health and disease prevention has become a key direction in academic research (Lee et al., 2018). Therefore, the research related to probiotics to enhance the immunity of the body is of great significance.

Different types of probiotic strains play different immunomodulatory roles and have different effects on immune cells and immunity (Yahfoufi et al., 2018). Complex probiotics that are made of different single strains of bacteria exhibited better effect on the regulation of immunity. For example, VSL#3 (Cheng et al., 2020), which was considered as medical food, contains eight live strains of bacteria, including four strains of *Lactobacillus*, three strains of *Bifidobacterium* and one strain of *Streptococcus*. *B*. *lactis* XLTG11 was isolated from the intestine of healthy infants with excellent probiotic properties, which can regulate the immunity system, alleviate inflammation, enhance intestinal barrier and regulate gut microbiota (Weiwei et al., 2022; Xu et al., 2022). It has been suggested that *Lactobacillus plantarum* P8 can survive in the host intestine, improve the community structure of intestinal flora, reduce the adhesion, infestation and colonization of harmful bacteria, promote the growth of beneficial bacteria, enhance the barrier function of intestinal flora, and have a positive regulatory effect on humoral immunity, cellular immunity and immunity of intestine-related lymphoid tissues (Wang et al., 2014). It has been shown that *Lacticaseibacillus casei* Zhang can increase the ratio of Th1 to Treg, and induces antibody class switching in activated B cells in the B cell immune response. Thereby expressing and producing IgG, IgA or IgE to exert immunomodulatory effects (Wang et al., 2013; Fu et al., 2020). Therefore, in this experiment, three strains with excellent probiotic properties were prepared into a complex probiotic powder to study their immunomodulatory effects on the host.

As important immune organs, the thymus and spleen indices, visual indicators of non-specific immunity, can reflect the immune function of the body (Zhao et al., 2020), which is consistent with the results of the present study, suggesting that probiotic complex can improve the immunity of CTX-damaged mice. CTX has an immunosuppressive effect on the intestinal mucosa, resulting in reduced intestinal villus height and increased crypt depth (Keefe et al., 2000; Owari et al., 2012). Our data showed that oral administration of the complex probiotic to mice increased the villi height (VH)/crypt depth (CD) ratio as well as the number of cupped cells, significantly attenuating CTX-induced intestinal epithelial damage, in agreement with other studies that a probiotic strain of *L. plantarum* isolated from pickled vegetables could effectively reverse the villus height and crypt depth changes from treatment with CTX (Xie et al., 2016). Goblet cells can regulate mucin synthesis and participate in the formation of intestinal mucosal barrier (Knoop and Newberry, 2018). Our results showed that the high dose of the complex probiotics treatment significantly increased ileum goblet cells, which was similar with the results of Park and Lee (2018).

Several studies have indicated that the leukocytes and lymphocytes counts increased significantly in mice treated with CTX by probiotic treatment (Lv et al., 2021; Wang et al., 2022), which is consistent with the results of the present study. CD3^+^ molecule is a marker of T lymphocytes (Li et al., 2016), CD4^+^CD8^−^ T lymphocytes are Th-type lymphocytes, which participate in the secretion of cytokines, promote the proliferation and differentiation of B lymphocytes, T lymphocytes and other immune cells, and play an innate immune response role. CD4^−^CD8^+^ T lymphocytes are Tc-type lymphocytes with specific killing effect and cytotoxicity, which can kill target cells (Li et al., 2019; Han et al., 2022). NK cells are potent effectors of the innate immune system and play a critical role in early defense against pathogens and foreign complex (Knoop and Newberry, 2018). The results of this study showed that the percentage of splenic lymphocytes subpopulation was reduced in CTX-treated mice and the percentage of splenic lymphocytes CD3^+^, CD4^+^CD8^−^, CD4^−^CD^8+^ and NK cells was increased in the complex probiotic group.

Cytokines are secreted by activated immune cells, which play an important regulatory role in the immune system. They can interact with each other to form a signal network and play a role in regulating the host immune level (Bai et al., 2022). IL-6 stimulates B cell proliferation, secretes antibodies, stimulates T cell proliferation and promotes blood cell development (Tanaka et al., 2018). It has been reported that IL-10 are closely related to mucosal sIgA production (Mantis et al., 2011). In T-cell immunity, IL-1β acts as an important mediator to promote the development, commitment, and effector function of T cells (Lasiglie et al., 2011). IFN-γ regulates adaptive immune responses *in vivo* by processing and presenting antigens, up-regulating pathogen recognition, participating in leukocyte transport, and inhibiting cell proliferation and apoptosis (Todorović-Raković, 2022). The experimental results showed that, with increasing doses of compound probiotics, the level of immune factors (IL-6, IL-10, IL-1β, IFN-γ), after being inhibited by cyclophosphamide, was significantly increased, leading to a subsequent improvement in the immune ability of the mice. Similar to the results of Lv et al. (2021), who enhanced the immunity of CTX-induced immunodeficient mice using a combination of live *Bifidobacterium*, *Lactobacillus*, *Enterococcus,* and *Bacillus*.

The results of this study showed that at the phylum level, Bacteroidetes decreased and Firmicutes increased in the CTX group compared to the CTRL group. The Bacteroidetes Firmicutes/Bacteroidetes phylum (F/B) ratio increased. This finding is consistent with previous reports of cyclophosphamide-induced alterations in the gut microbiota (Xu and Zhang, 2015; Zhang et al., 2020) and may be due to CTX -induced immunodeficiency. Interestingly, the complex probiotic treatment, especially in the CM, CH group, alleviated these imbalances and reduced the proportion of the Firmicutes/Bacteroidetes, which is considered a good indicator of significant changes in microbial composition (Chen et al., 2022; Xie et al., 2022). In addition, at the genus level, CTX reduced the relative abundance of probiotic bacteria such as *Bacteroides*, *Prevotellaceae*_NK3B31, *Prevotellace*ae_UCG-001, *Clostridia*, *Odoribacter*, while the complex probiotics treatment significantly inhibited this trend, which was similar to the results of Zhang et al. (2020), who reported that *Lactobacillus plantarum* BF_15 isolated from the feces of breastfed infants could effectively alleviate CTX-induced immunosuppression by rebalancing the gut microbiota. Compared to the CTX group, the complex probiotics improved the abundance of *Bacteroides*, *Alloprevotella*, *Alistipes,* and *Lachnospiraceae*_NK4A136_group. *Bacteroides* and *Alloprevotella* are short chain fatty acids (SCFAs)-producing bacteria and anti-inflammatory bacteria (Han et al., 2022). The secretion of SCFAs by *Bacteroides* species can prevent the transport of toxins between the gut lumen and blood, and colon tumor formation in humans (Wang et al., 2022). *Alloprevotella* can produce SCFAs, mainly succinate and acetic acid, which could improve the intestinal epithelial barrier and prevent inflammation (Downes et al., 2013). In addition, the *Lachnospiraceae*_NK4A136_group are the SCFAs producer in gut microbiota with potential ulcerative colitis -ameliorating properties (Shi et al., 2017; Wang et al., 2019). Accumulated evidences indicated that *Alistipes* may have protective effects against some diseases, including liver fibrosis, colitis, cancer immunotherapy, and cardiovascular disease (Parker et al., 2020). It was confirmed that the complex probiotic could restore the gut microbiota imbalance in CTX -induced immunosuppressed mice.

## 5. Conclusion

In conclusion, the present study showed that the complex probiotics could improve the spleen and thymus indices and the intestinal morphology. The leukocyte and lymphocyte counts and the percentage of splenic lymphocyte subpopulations in the CTX-treated mice were elevated by the complex probiotics. In addition, the cytokines (IL-6, IL-10, IL-1β, and IFN-γ) were increased after complex probiotic treatment. The complex probiotics could restore the disordered gut microbiota. This study provides theoretical support for the immunity-enhancing function of a complex microbial preparation composed of *B. lactis* XLTG11, *L. casei* Zhang, and *L. plantarum* P8, as well as a pharmacological basis for its further development and utilization.

## Data availability statement

The datasets presented in this study can be found in online repositories. The names of the repository/repositories and accession number(s) can be found at: https://www.ncbi.nlm.nih.gov/sra/, PRJNA884281.

## Ethics statement

All animal procedures were performed in accordance with the Guidelines for Care and Use of Laboratory Animals of Heilongjiang Chinese Medicine University and the experiments were approved by the Animal Ethics Committee of Heilongjiang Chinese Medicine University (ethic approval code: 2021121201).

## Author contributions

LH and WM designed the study. QM and ZZ performed the experiments. WM, WL, and SY wrote the manuscript. HB, YW, and YJ analyzed the data. All authors contributed to the article and approved the submitted version.

## Funding

Present research work was financially supported by grants from Natural Science Foundation of Heilongjiang Province of China (Nos. LH2019H106 and LH2020H098), Doctoral Innovation Research Fund of Heilongjiang University of Traditional Chinese Medicine (No. 2015bs08), National Natural Science Foundation of China (Nos. 81274114 and 82003974), Heilongjiang Province Touyan Team, Key Laboratory of the Ministry of Education--North Medicine Fundamental and Applied Research Open Fund.

## Conflict of interest

The authors declare that the research was conducted in the absence of any commercial or financial relationships that could be construed as a potential conflict of interest.

## Publisher’s note

All claims expressed in this article are solely those of the authors and do not necessarily represent those of their affiliated organizations, or those of the publisher, the editors and the reviewers. Any product that may be evaluated in this article, or claim that may be made by its manufacturer, is not guaranteed or endorsed by the publisher.
